# Polydopamine-Coated Liposomes for Methylene Blue Delivery in Anticancer Photodynamic Therapy: Effects in 2D and 3D Cellular Models

**DOI:** 10.3390/ijms25063392

**Published:** 2024-03-16

**Authors:** Vincenzo De Leo, Emanuela Marras, Anna Maria Maurelli, Lucia Catucci, Francesco Milano, Marzia Bruna Gariboldi

**Affiliations:** 1 Department of Chemistry, University of Bari Aldo Moro, via Orabona 4, 70126 Bari, Italy; vincenzo.deleo@uniba.it (V.D.L.); anna.maurelli@uniba.it (A.M.M.); lucia.catucci@uniba.it (L.C.); 2 Department of Biotechnology and Life Sciences (DBSV), University of Insubria, via JH Dunant 3, 21100 Varese, Italy; emanuela.marras@uninsubria.it; 3 Institute of Sciences of Food Production, Italian National Research Council (CNR-ISPA), S.P. Lecce-Monteroni, I-73100 Lecce, Italy; francesco.milano@cnr.it

**Keywords:** methylene blue, liposome, polydopamine, photodynamic therapy, 2D and 3D cellular models

## Abstract

Photodynamic therapy (PDT) is a therapeutic option for cancer, in which photosensitizer (PS) drugs, light, and molecular oxygen generate reactive oxygen species (ROS) and induce cell death. First- and second-generation PSs presented with problems that hindered their efficacy, including low solubility. Thus, second-generation PSs loaded into nanocarriers were produced to enhance their cellular uptake and therapeutic efficacy. Among other compounds investigated, the dye methylene blue (MB) showed potential as a PS, and its photodynamic activity in tumor cells was reported even in its nanocarrier-delivered form, including liposomes. Here, we prepared polydopamine (PDA)-coated liposomes and efficiently adsorbed MB onto their surface. lipoPDA@MB vesicles were first physico-chemically characterized and studies on their light stability and on the in vitro release of MB were performed. Photodynamic effects were then assessed on a panel of 2D- and 3D-cultured cancer cell lines, comparing the results with those obtained using free MB. lipoPDA@MB uptake, type of cell death induced, and ability to generate ROS were also investigated. Our results show that lipoPDA@MB possesses higher photodynamic potency compared to MB in both 2D and 3D cell models, probably thanks to its higher uptake, ROS production, and apoptotic cell death induction. Therefore, lipoPDA@MB appears as an efficient drug delivery system for MB-based PDT.

## 1. Introduction

Photodynamic therapy (PDT) is a relatively new therapeutic approach to cancer treatment in which, under light irradiation at specific wavelengths, photosensitizer molecules (PSs) react with molecular oxygen and generate reactive oxygen species, ultimately killing cancer cells [1,2]. Specifically, the sensitized PS produces hydrogen peroxide, superoxide anion radical, hydroxyl radical (type I mechanism), or singlet oxygen (type II mechanism) through electron or proton transfer [3]. In clinical practice, PSs are administrated locally or systemically and accumulate in the target tissues which are the only ones to be irradiated, thus reducing extra toxicity or injuries to the structures near the tumor [1,4]. In superficial lesions, the irradiation is direct, while a fiber optic catheter must be used to obtain exposure to the target when internal organs are involved [5,6]. Due to their cost-effectiveness and easy handling, diode lasers are commonly used as a light source for PDT [5].

Direct killing of tumor cells, vasculature damage induction, and stimulation of the immune system are the interrelated mechanisms involved in the antitumor effects of PDT [2,7]. Several conventional cell death pathways are implicated in the specific mechanisms through which PDT induces cell death, mainly apoptosis, necrosis, and autophagy [1,8]. Nevertheless, non-conventional cell death modalities, such as pyroptosis, necroptosis, and ferroptosis, have been related to PDT-cytotoxic effects [9].

PSs play a pivotal role in the efficacy of PDT [7]. First-generation PS hematoporphyrin derivatives were the first to be approved for clinical use [10,11]. Still, they presented limitations to their use due to side effects, such as photosensitization and poorly penetrating low-absorption wavelengths. Second-generation PSs showed improved physicochemical features but also had limited clinical use [10,12]. Third-generation PSs, consisting of second-generation PSs conjugated with targeting moieties or loaded into nanoparticles, are currently being developed to improve efficacy [13,14]. In addition, other chemical classes of PSs are under investigation, including BODIPYs, dyes such as Rose Bengal, methylene blue (MB), Toluidine blue, and acridines. Optimal absorption in the 600–900 nm window, low toxicity in the absence of light (dark), absence of toxic secondary metabolites, selective accumulation in cancer cells and specific targeting of cellular organelles, low rate of photobleaching, optimal absorption, distribution, and excretion are all characteristics sought after in the ideal PS. Among others, MB has been shown to meet some of these desirable features. Originally used as a dye and later for treating various pathological conditions [15,16,17,18], MB has also emerged as a promising PS for PDT. As a matter of fact, due to the phenothiazine chromophore, MB absorbs the light in the wavelength range from 630 to 680 nm and experimental evidence indicates in vitro and in vivo activity against several types of tumors [19,20,21]. Furthermore, MB induces the formation of both radical and singlet oxygen species, thus extending its application to tumors with hypoxic areas in which the type II mechanism does not occur efficiently [21,22].

Ensuring a sufficient PS delivery to the target tissues can enhance the efficacy of PDT and reduce side effects. Thus, several nanomaterials have been studied as PS carriers to allow the specific passive or active delivery of PS and enhance the efficacy of irradiation on the targeted tissue, leading to the improved clinical potential of PDT [15,23]. Interestingly, nanoparticles can also preserve the PSs from photobleaching [24]. Among the different nanocarriers, liposomes present some advantages such as low toxicity, biodegradability, and biocompatibility due to their lipid nature [25]. Liposomes consist of a self-assembled bilayer of phospholipids enclosing an aqueous core, resulting in vesicles capable of encapsulating hydrophilic and hydrophobic molecules [26,27,28]. The characteristics of liposomes can be tuned to meet specific application needs. This can be achieved by varying the lipid composition, adding polymer coatings, or introducing specific targeting functionalities [29,30].

Liposomes have shown great potential in increasing drug permeability into biological membranes, as well as allowing their release in a sustained way [31,32]. Experimental evidence indicates that PSs encapsulated into liposomes suppress the growth of several types of cancer, such as breast cancer, biliary tract cancer, and gastric cancer [33,34,35], as well as reducing the dark toxicity of the PS and improving cellular uptake [36,37]. In particular, different MB preparations encapsulated in nanoparticles, even liposomes, have been tested and have shown better results than the non-encapsulated dye [15,20,38]. This is a great opportunity, as some works demonstrated that MB in liposomes could be used as an antibacterial agent and for the PDT of cancer cells [15,20,26]. Among others, developments in MB nanomaterials for PDT applications have recently emerged [39,40,41].

In a previous work, we demonstrated that polydopamine (PDA)-coated liposomes can efficiently adsorb MB due to favorable electrostatic interactions [42]. PDA is a synthetic polymer easily obtainable as a result of the polymerization of dopamine (DA) under weakly alkaline conditions. This polymer presents several functional groups, such as catechol and amino groups and aromatic moieties, that can bind a variety of compounds through covalent and non-covalent interactions [43]. Moreover, PDA is generally recognized as a biocompatible and biodegradable material [44]. In this work, we prepared PDA-coated small unilamellar vesicles (lipoPDA) and adsorbed the MB onto their surface. Thus, we tested the obtained lipoPDA@MB vesicles as a third-generation PS agent for anticancer PDT. The physicochemical characterization of the vesicles was performed first, along with the quantification of the in vitro release of MB from the vesicles and their light stability. The photodynamic effects of the vesicles were then evaluated in a panel of monolayer- and 3D-cultured cancer cell lines, comparing the results with those obtained by free MB. Fluorescent Rhodamine-lipoPDA vesicles were also used to evaluate the uptake of the nanoparticles into 2D- and 3D-cultured cell lines, and MB and lipoPDA@MB intracellular accumulation has also been assessed. Furthermore, the most common type of cell death mechanisms related to PDT were investigated, together with the ability of both formulations to induce ROS and singlet oxygen production.

## 2. Results

### 2.1. Liposome Preparation and Characterization

In this work, uncoated small unilamellar liposomes were prepared with the so-called Micelle-To-Vesicle Transition (MVT) method (see Section 4.2 for methodology, acronyms, and equations), and then incubated with DA at pH 8.0 to obtain lipoPDA with final dimensions below 100 nm. Both uncoated and PDA-coated liposomes were characterized from a colloidal standpoint by means of Dynamic Light Scattering (DLS) and ζ-potential analysis (Table 1). DLS measurements showed that the initial diameters of the liposomes were around 25 nm and increased after the PDA coating to about 53 nm. The PDA shell thickness was estimated as the difference between the final and starting radius and was around 14 nm. The polydispersity index (PDI) was 0.18 for uncoated liposomes and remained below 0.3 after PDA coating, as required for particles used in biomedical applications. TEM images revealed circular-shaped structures with dimensions in good agreement with the DLS data (see Supplementary Figure S1). The size and contrast of liposomes were higher after PDA grafting, as previously observed for lipoPDA vesicles [42]. The analysis of the ζ-potentials at pH 7.0 indicated the existence of negative surface charges on the vesicles. It is reported that PDA possesses an isoelectric point at pH values around 4 and shows positive or negative charges below or above this value, respectively [42,43]. Under near-neutral pH conditions, electrostatic forces are key in promoting interactions between the vesicles and the photosensitizer, as MB is cationic and the vesicles are negatively charged.

lipoPDA@MB vesicles were therefore obtained by incubating the lipoPDA liposomes in an aqueous solution of MB, leaving the photosensitizer to adsorb onto the PDA coating. At the end of the process, the system was purified of excess unbound MB by size exclusion chromatography (SEC). Table 1 shows a moderate increase in the final size of the liposomal system after MB adsorption, and the size distribution of lipoPDA@MB is almost monodisperse, while the TEM images showed no appreciable changes in morphology compared to lipoPDA vesicles (Figure S1). The amount of MB adsorbed onto lipoPDA@MB vesicles, estimated after solvent extraction with EtOH, was about 134 μM, with an EE% of 24 ± 3 and a LC% of 0.9 ± 0.2. The LC was similar to the one previously estimated for MB loaded in the aqueous core of liposomes [26] but with the advantage of reduced overall carrier size (60 nm vs. ≈150 ÷ 240 nm [26,38]), which may result in better cellular uptake.

**Figure 1:**
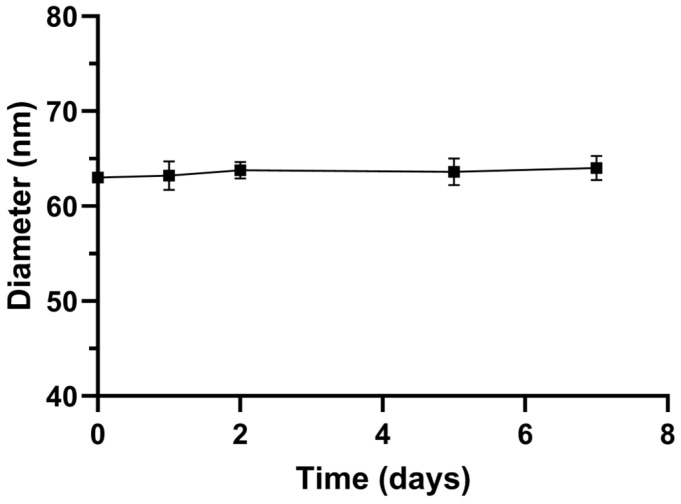
Colloidal stability of lipoPDA@MB stored in phosphate buffer pH 7.0 at 4 °C in terms of mean diameter variations over time. Data are expressed as mean ± SD (n = 3).

The stability of the lipoPDA@MB vesicles in PBS was estimated by measuring their size variations using DLS analysis. Over seven days, negligible changes in average diameter were measured (Figure 1), as the PDA coating provides steric stabilization to the liposomes similar to other polymer coatings such as PEG ones.

### 2.2. In Vitro Release Assay

**Figure 2:**
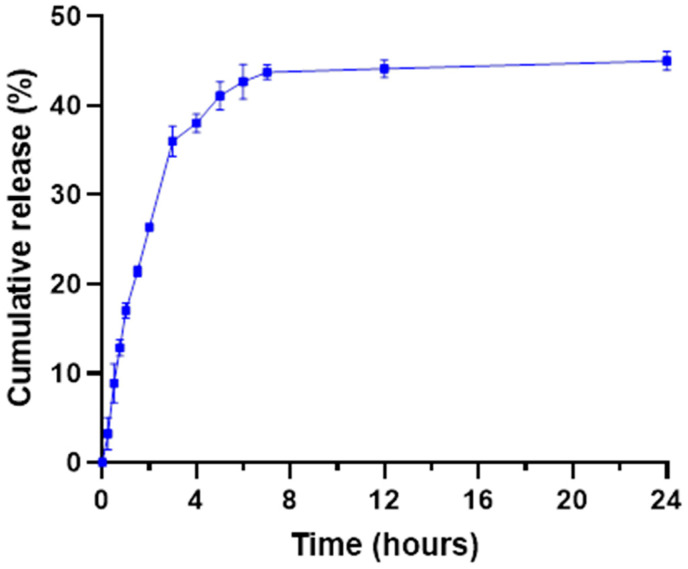
Cumulative in vitro release of MB from lipoPDA@MB vesicles in PBS 1X, pH 7.4. The error bars represent the experiment’s standard deviation (S.D.) (*n* = 3).

The in vitro cumulative release of MB from lipoPDA@MB vesicles was assessed at physiological pH in PBS buffer through a dialysis-based method. Figure 2 shows a fast release of the PS in the first 4 h, followed by a subsequent slower release, which within about 7 h reached a plateau of around 45% of MB released, with virtually no further variations up to 24 h. Thus, the slow release achieved by loading the PS onto the PDA shell could be a useful strategy to avoid its loss in the organism before reaching the tumor site.

### 2.3. Photostability

**Figure 3:**
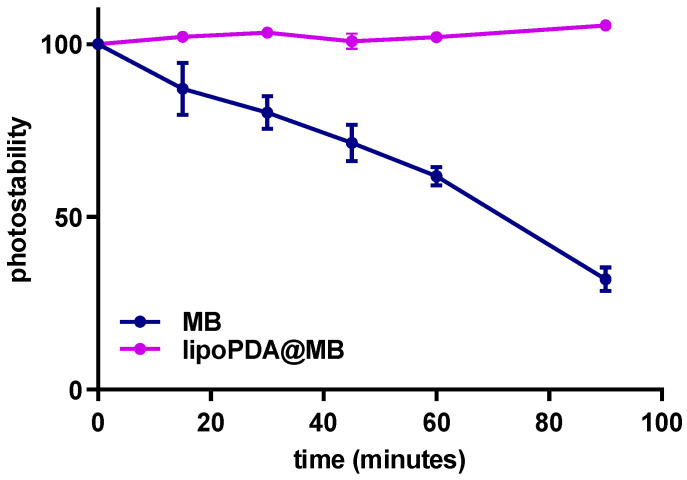
Photostability of MB and lipoPDA@MB during 90 min irradiation with a halogen light (500 W, irradiance 22 mW/cm^2^). Values reported are the means ± S.D of three replicates.

Photodegradation of MB was evaluated by measuring the decrease in absorbance intensity during 90 min irradiation of MB and lipoPDA@MB with a white tungsten halogen light (500 W, irradiance 22 mW/cm^2^; fluence of 100 J/cm^2^) to determine their photostability. Figure 3 reported photostability percentages calculated at each time point as the ratio of absorption intensity to absorption measured at the t0.

As shown in Figure 3, photobleaching of free MB was much faster than that of lipoPDA@MB, indicating that the process of photodegradation was less effective in the latter than in the solution of MB.

### 2.4. Effect on Cell Viability and Cell Growth

**Figure 4:**
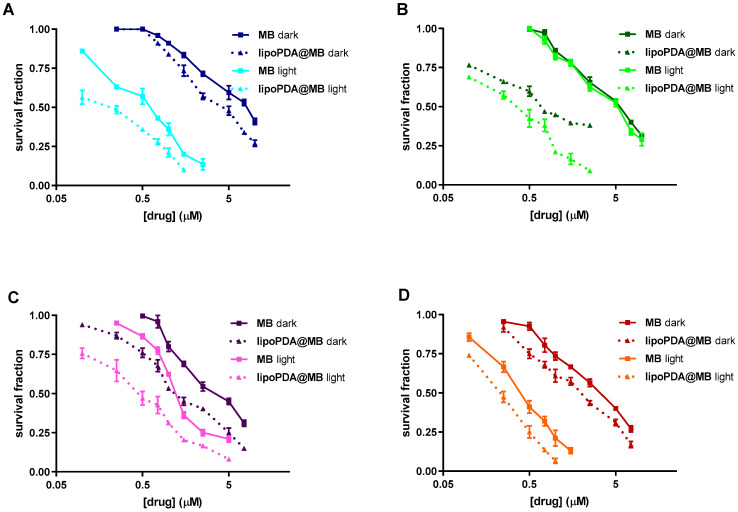
Dose-response curves obtained following 24 h treatment of HCT116 (**A**), HT29 (**B**), MCF7 (**C**), and MDA-MB231 (**D**) cells with MB and lipoPDA@MB, 1 h irradiation, 24 h incubation in drug-free medium, and MTT assay (mean ± S.D. of 3–4 independent experiments).

The MTT assay was performed to evaluate the photodynamic activity of MB and lipoPDA@MB on HCT116, HT29, MCF7, and MDA-MB231 cell lines grown in monolayers. To this aim, cells were treated with increasing concentrations of the two formulations, irradiated for 1 h, and incubated for 24 h in a drug-free medium. The obtained dose-response curves are represented in Figure 4 and the corresponding IC_50_ values are reported in Tables S1 and S2. To evaluate the intrinsic cytotoxicity of the photosensitizers, the irradiation step was omitted from the treatment protocol (dark). In this condition, the tested formulations showed cytotoxic effects at micromolar concentrations. Furthermore, in all cell lines, lipoPDA@MB was significantly more potent than MB (from 1.3-fold for HCT116 cells to 2.9-fold for HT29 cells), as indicated by the left shift of the dose-response curves and the lower IC_50_ values (Tables S1 and S2). Interestingly, in all the tested cell lines, PDT significantly increased the potency of both lipoPDA@MB and MB, resulting in submicromolar IC_50_ values. The lower increment was observed in HCT116 cells (two-fold), the higher in HT29 cells (eight-fold). Moreover, lipoPDA@MB was still significantly more potent than MB. Furthermore, no MB and lipoPDA@MB photodynamic effects were observed in the fibroblast cell line WH1, used as a model of normal cells, treated in the same conditions used for the tumor cell lines (Figure S2), indicating a selectivity of the tested compounds for tumor cells.

The possible toxic effects of lipoPDA vesicles were also evaluated by treating the four cell lines with lipoPDA dilutions equal to those used for lipoPDA@MB, and the IC_50_ values obtained, expressed as MB-equivalent, were higher than 5 μM in all cases (Tables S1 and S2).

**Figure 5:**
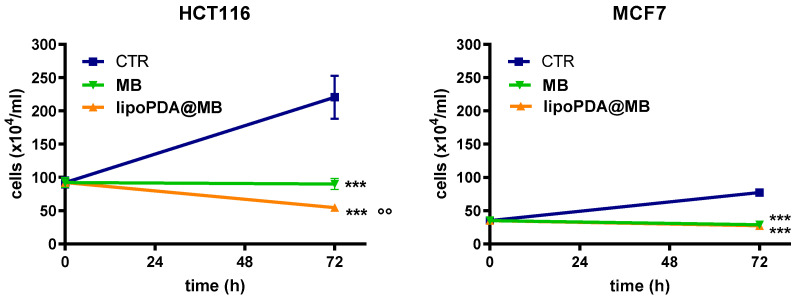
Growth of HCT116 and MCF7 spheroids following 24 h treatment with MB and lipoPDA@MB at concentrations corresponding to the IC_50_ values obtained in monolayer-cultured cells, 1 h irradiation, and 24 h incubation in a drug-free medium in the dark. Counts of viable cells were performed immediately following irradiation (time 0) and 72 h later (mean ± S.D. of 3/5 spheroids; *** *p* < 0.001 vs. C; °° *p* < 0.01 vs. MB).

The effects of free and lipoPDA-loaded MB were also evaluated in spheroids obtained from HCT116 and MCF7 cells. Specifically, spheroid growth and cell viability were evaluated following 24 h of treatment with MB and lipoPDA@MB at concentrations corresponding to IC_50_ values obtained in monolayer-cultured cells, 1 h irradiation, and incubation in a drug-free medium in the dark. The results are reported in Figure S3 and Figure 5. Pictures of the spheroids were taken through a camera connected to an Olympus IX8I microscope following irradiation (0), and 72 h later. At the same time, 3/5 spheroids were collected, disaggregated, and live cells were counted based on a dye exclusion assay. The growth curves were drawn (Figure 5).

HCT116 spheroids grew faster than MCF7 ones, as indicated by the bigger size observed in the control (Figure S3). Treatment with both formulations, followed by photoactivation, resulted in a significant reduction of spheroid size compared to control spheroids. In particular, many cells were released from the external layers of spheroids, following treatment and PDT, especially after treatment with lipoPDA@MB. Results from the Trypan blue-exclusion cell count assay (Figure 5) are in agreement with the images shown and indicate that the number of HCT116 and MCF7 viable cells found in control (i.e., untreated) spheroids at the end of the incubation period are higher than those at t0 (92.33 ± 8.3 × 10^4^ cells/mL and 35.1 ± 3.98 × 10^4^ cells/mL in HCT116 and MCF7 cells, respectively). Furthermore, following treatment with MB and lipoPDA@MB, a significant decrease in cell number was observed in both cell lines compared to the control. Interestingly, in HCT116 spheroids, lipoPDA@MB was confirmed to be significantly more potent than MB.

### 2.5. Intracellular Accumulation

The intracellular accumulation of the vesicles in HCT116, HT29, MCF7, and MDA-MB231 cells after 24 h of incubation was first evaluated through flow cytometry, by exploiting the fluorescence of lipoPDA vesicles containing a Rhodamine-functionalized phospholipid (Rhodamine-lipoPDA), which have a size and surface charge analogous to lipoPDA (Table S3). Preliminary results have shown that the median fluorescence intensity (MFI) observed for lipoPDA-treated cells was generally low and similar to that of controls (untreated, CTR) in all cell lines. On the other hand, Rhodamine-lipoPDA vesicles entered all the cell lines at higher extents. Specifically, in colorectal cancer, a higher accumulation of liposomes was observed in HCT116 cells compared to HT29 cells. Concerning breast cancer, the accumulation in MCF7 and MDA-MB231 cell lines was comparable (Figure S4).

**Figure 6:**
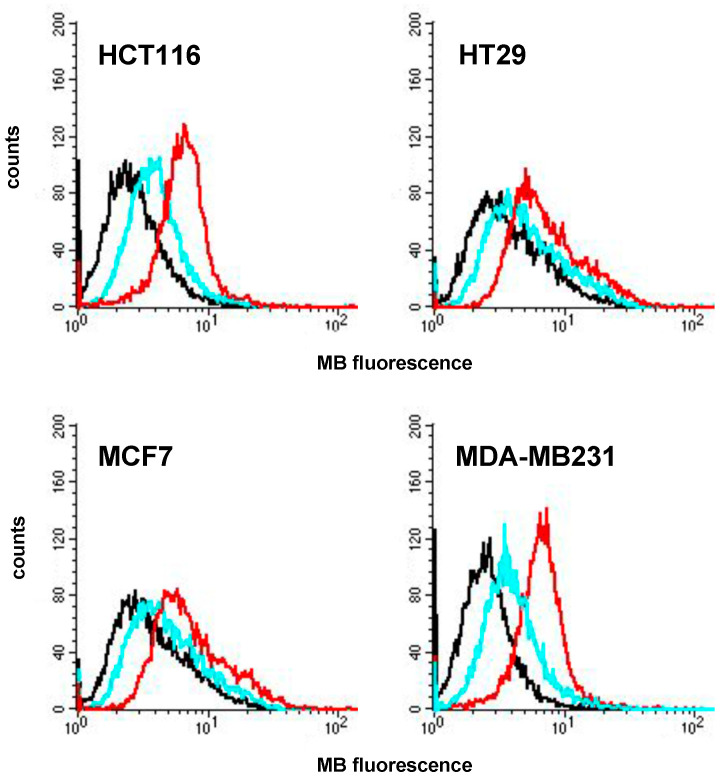
Intracellular uptake of MB and lipoPDA@MB (controls: black line; MB: light blue line; lipoPDA@MB: red line).

MB and lipoPDA@MB cellular uptake was then assessed through flow cytometry by exploiting MB fluorescence. Similar results to those shown in Figure S4 were obtained and reported in Figure 6. Interestingly, the same trend was evidenced in all cell lines, as higher MB fluorescence levels were observed in lipoPDA@MB-treated cells than in MB-treated ones, indicating that lipoPDA@MB vesicles entered all the cell lines better than free MB. However, as evidenced in the preliminary accumulation experiments, HCT116 and MDA-MB231 cells accumulate lipoPDA nanoparticles at a higher extent than HT29 and MCF7 cells.

**Figure 7:**
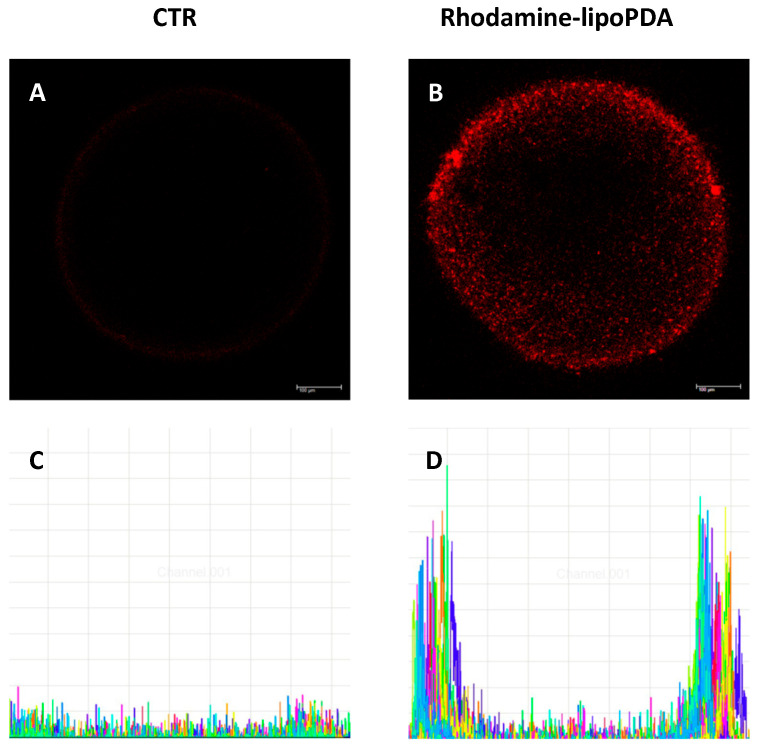
Penetration of Rhodamine-lipoPDA (100 nM) in HCT116 spheroids after 24 h incubation. Pictures show Rhodamine fluorescence at the equatorial plane of the spheroids (**A**,**B**), while histograms represent the analysis of distribution and intensity of the Rhodamine fluorescence in 15 different randomly traced diameters in the equatorial planes, which are represented in different colors (**C**,**D**).

Confocal microscopy images obtained on HCT116 spheroids (Figure 7A,B) and the analysis of the distribution and intensity of PS fluorescence in the images of the equatorial planes (Figure 7C,D) indicate that lipoPDA vesicles also enter 3D-cultured cells. Furthermore, penetration of Rhodamine-lipoPDA vesicles was limited to the external cell rim of the spheroids, while in their inner core, only low fluorescence was detected, meaning lower penetration of the vesicles in that part of spheroids.

### 2.6. Cell Death Induction

The type of death induced by MB and lipoPDA@MB vesicles, in the dark or following PDT, was investigated through flow cytometric analysis of HCT116, HT29, MCF7, and MDA-MB231 cells and HCT116 and MCF7 spheroids treated with the equitoxic concentrations of the two formulations corresponding to the respective IC_50_ values reported in Tables S1 and S2.

**Figure 8:**
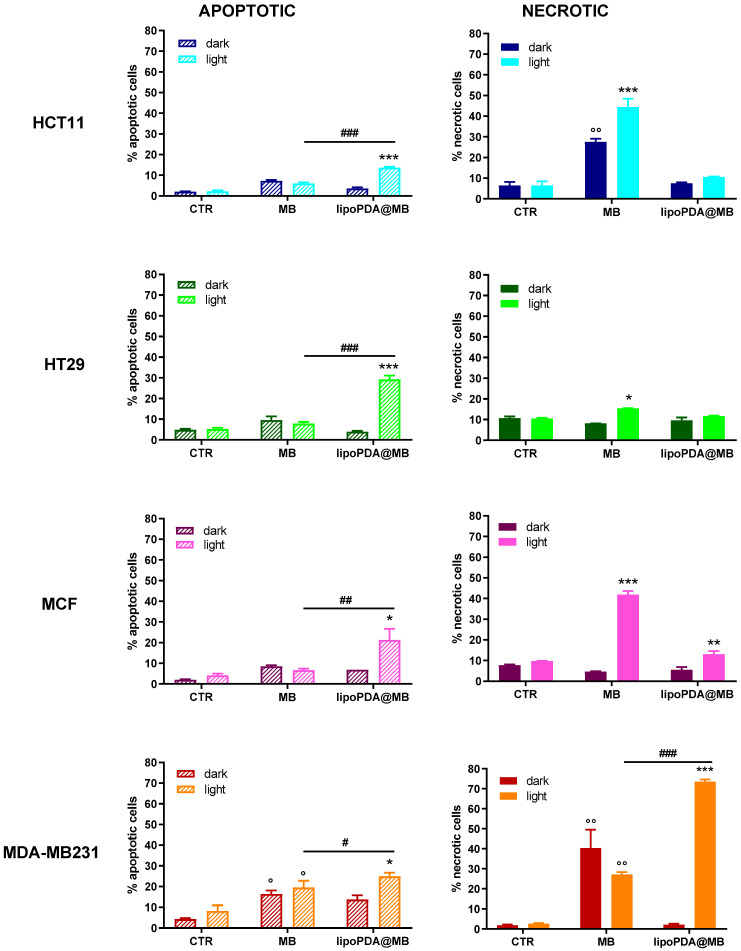
Percentages of apoptotic and necrotic HCT116, HT29, MCF7, and MDA-MB231 cells, following 24 h treatment with MB and lipoPDA@MB at equitoxic concentrations corresponding to the respective IC_50_ values, 1 h irradiation (500 W, irradiance 22 mW/cm^2^; fluence of 100 J/cm^2^), 24 h incubation in drug-free medium. Propidium iodide was used as a DNA probe (mean ± SE of 3 independent experiments; ° *p* < 0.05 and °° *p* < 0.01 vs. CTR; * *p* < 0.05, ** *p* < 0.01 and *** *p* < 0.001 vs. CTR and dark; # *p* < 0.05, ## *p* < 0.01, ### *p* < 0.001).

When the cells were kept in the dark, a significant increase in necrotic cell death, over the controls, was observed only in MB-treated MDA-MB231, while both MDA-MB231 and HCT116 cells underwent apoptotic cell death in the same condition. On the contrary, the lipoPDA@MB vesicles were not able to induce apoptotic or necrotic cell death in the dark (Figure 8).

Following PDT, apoptosis was a major contribution in the cells treated with lipoPDA@MB; only MDA-MB231 cells responded to MB treatment by increasing the percentage of apoptotic cells. Interestingly, the percentages of apoptotic cells observed were significantly higher in the lipoPDA@MB-treated cells compared to MB-treated ones. Concerning necrosis, this type of cell death was preferentially induced by MB in all cell lines; only in MCF7 and MDA-MB231 cells did lipoPDA@MB vesicle treatment result in an increased percentage of necrosis (Figure 8).

**Figure 9:**
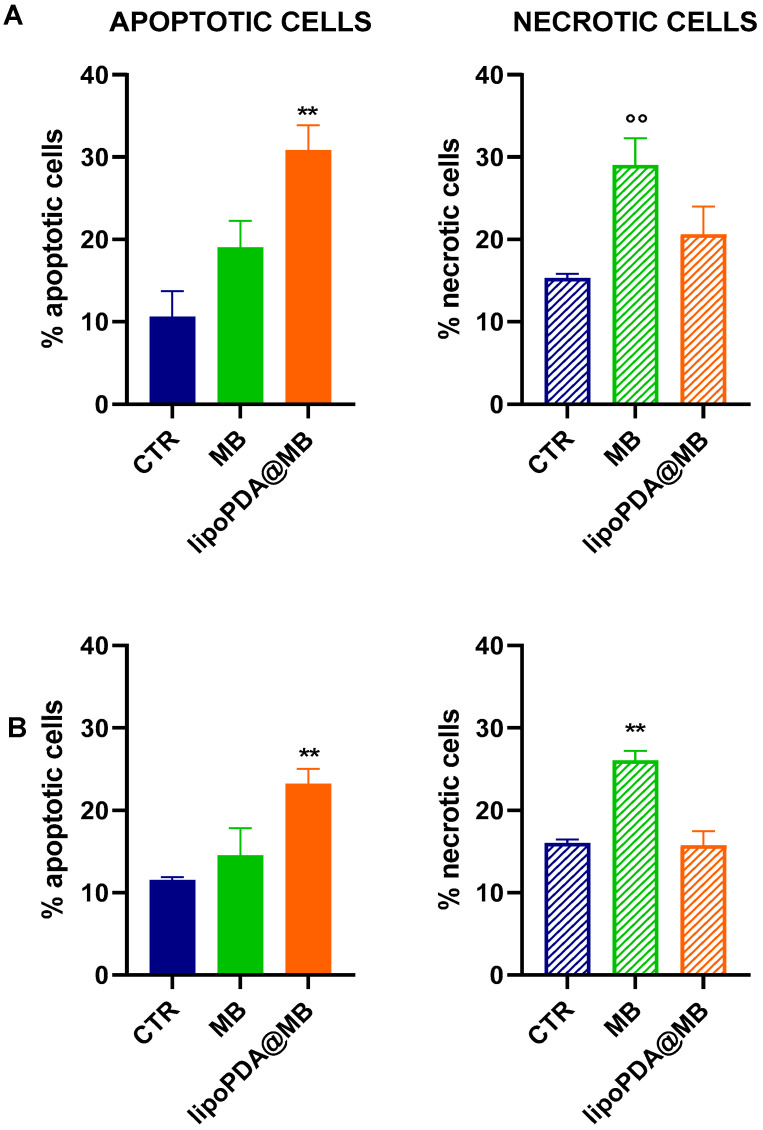
Percentages of apoptotic and necrotic cells obtained from HCT116 (**A**) and MCF7 (**B**) spheroids following 24 h incubation with MB and lipoPDA@MB at concentrations corresponding to the IC_50_ values obtained in monolayer-cultured cells, 1 h irradiation (500 W, irradiance 22 mW/cm^2^; fluence of 100 J/cm^2^), and 72 h incubation in a drug-free medium. Propidium iodide was used as a DNA probe (mean ± S.D. of 3 independent experiments in which 3/5 spheroids/treatment were used; °° *p* < 0.01 vs. CTR; ** *p* < 0.01 vs. CTR and MB).

The ability of MB and lipoPDA@MB to induce apoptotic cell death was also evaluated in spheroids obtained from HCT116 and MCF7 cells following 24 h incubation with the two formulations, 1 h irradiation, and 72 h incubation in a drug-free medium. In this 3D model, MB and lipoPDA@MB preferentially induced necrotic and apoptotic cell death, respectively (Figure 9).

### 2.7. Evaluation of ROS and Singlet Oxygen Production

**Figure 10:**
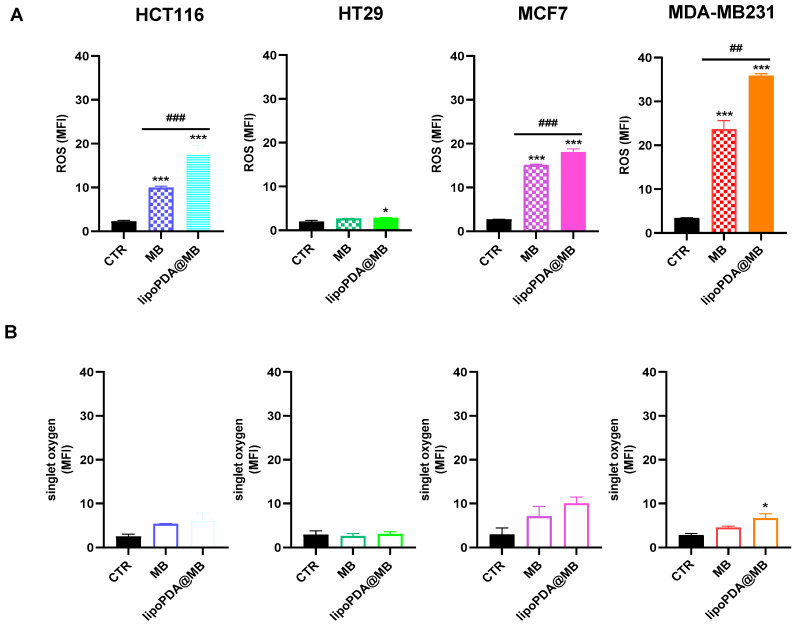
ROS (**A**) and singlet oxygen (**B**) production in HCT116, HT29, MCF7, and MDA-MB231 cell lines following incubation with equitoxic concentrations of MB and lipoPDA@MB, corresponding to their respective IC_50_ values, incubation with H2DCF-DA or siDMA and 2 min irradiation (500 W, mean ± S.D. of 3 independent experiments; * *p* < 0.05 and *** *p* < 0.001 vs. CTR; ## *p* < 0.01 and ### *p* < 0.001).

Figure 10 shows intracellular ROS (A) and singlet oxygen levels (B) evaluated using H2DCF-DA and siDMA as probes, respectively. Furthermore, Figure S5 shows original example images of flow cytometric analysis of intracellular ROS. A significant increase in ROS production was observed following 24 h treatment with free MB and lipoPDA@MB vesicles in HCT116, MCF7, and MDA-MB231 cell lines, as indicated by the right shift of the fluorescein peaks in Figure S5. In agreement with cell viability results, in the three cell lines, lipoPDA@MB treatment induced a higher extent of ROS production compared to those observed in MB-treated cells. In HT29 cells, ROS levels were lower compared to the other cell lines; however, a significant increase was evidenced only following treatment with lipoPDA@MB.

Concerning singlet oxygen, a significant increase in its production was induced only by lipoPDA@MB treatment in MDA-MB231 cells.

## 3. Discussion

Due to the interest gained by anticancer photodynamic therapy (PDT), compounds for photosensitizing applications (namely, photosensitizers, PSs) have been broadly explored in the past few decades [7,45]. In searching for the ideal PSs, which should possess low dark cytotoxicity and the capacity to foster cytotoxicity when submitted to light, a high quantum yield of ^1^O_2_ and ROS, and specific tumor accumulation, three generations of PSs have been developed over the years [7,10]. To achieve a better efficacy of PDT, non-porphyrin-like PSs have also been investigated, including dyes such as Rose Bengal, phenothiazinium dyes (Methylene Blue (MB), Toluidine Blue), and acridines [10,11,21]. In particular, due to the phenothiazine group chromophore, MB has shown an interesting photosensitizing action [20,46]. Furthermore, MB selectively accumulates in cancer cells, making it a potential photosensitizer for anticancer PDT [20].

To improve PS efficacy, nanotechnology has recently emerged to ensure adequate delivery of the PSs to the target tissues, reducing side effects [6].

Recently, encapsulation of PS into liposomes was shown to be effective in suppressing the growth of some types of cancer [19,26]. Liposomes are considered outstanding candidates for the delivery of PSs in cancer PDT due to their ability to hold hydrophilic PSs in the aqueous interior and hydrophobic PSs in the lipid bilayer [19]. Several liposome-MB formulations have been successfully used in PDT [15,47].

A new formulation with improved absorption performances for MB has recently been obtained by our group by exploiting the outstanding adsorption properties of PDA [42].

In the present work, lipoPDA@MB vesicles were obtained by coating the liposomes with PDA and then by absorbing the photosensitizer molecules on the outer surface of the polymer coating. As previously described, the DA self-polymerization reaction in the presence of the liposomes under slightly alkaline pH conditions easily leads to the formation of a stable and uniform PDA coating on the surfaces of the lipid vesicles [42]. Unlike the previously prepared liposomal systems incorporating MB inside their aqueous core [26,38,41], here the EE% did not depend on the internal volume of the liposomes and it was possible to prepare a delivery system of reduced dimensions (<100 nm), with improved colloidal stability and better cellular uptake.

Interestingly, the slower release achieved by loading the photosensitizer onto the PDA shell instead of into the aqueous core of bare liposomes [26] could represent a useful strategy to avoid the loss of the PS in the organism before reaching the tumor site. However, at lower pH values, such as those found in in vivo conditions at tumor tissues [48,49], a more intense release of the photosensitizer could be triggered because of the decrease in the negative charge on the surface of the PDA coating, leading to a lower electrostatic interaction with the cationic MB payload.

Our results demonstrated a strong contribution of PDT action to cell death, induced by both free and MB-loaded lipoPDA. However, when delivered by liposomes, significantly enhanced toxicity of MB was observed, probably attributable to the superior ability of liposomes to interact with cells and to convey the PS inside them, as already reported [15,19]. In this regard, a significant uptake of rhodamine-labeled lipoPDA, MB, and lipoPDA@MB has been observed in the four cell lines studied, with higher intracellular accumulation assessed in HCT116 and MDA-MD231 cells. We did not specifically address the exact reasons for this behavior. Nevertheless, liposomes are known to enter cells by endocytosis, which can be mediated through proteins such as clathrin or caveolin among others, which may influence liposome uptake [50].

The less pronounced, but still significant, increment of the toxicity of lipoPDA@MB vesicles compared to MB, in dark conditions, was not a surprise. As a matter of fact, other authors have previously observed MB’s intrinsic non-photodynamic toxicity [51,52] and, although lipoPDA vesicles do not demonstrate toxic effects, the improved delivery could also justify the higher dark toxicity of the lipoPDA@MB formulation.

Photochemical stability is one of the most important parameters determining the usefulness of organic dyes in different applications, such as PDT [53]. Photobleaching of MB in aqueous solutions is a photodynamic process in which active oxygen species (ROS and singlet oxygen ^1^O_2_) generated during MB illumination can attack the sensitizer itself, leading to the so-called self-sensitized photo-oxidation [54]. Endoperoxide is one of the main products of the photo-oxidation reaction [55]. Previous observations showed that MB degrades relatively quickly when exposed to light [24,56]. In agreement, the process of photodegradation was more effective for MB than for the lipoPDA@MB vesicles, thus potentially enhancing the effectiveness of the latter in comparison to MB alone following PDT.

Several pieces of evidence established that the transition from 2D results to in vivo studies often resulted in a drastic reduction in PS activity [57,58] due to the inability of monolayer-cultured cells to accurately mimic the natural structures of tumors and the in vivo response to drugs [59,60]. However, lipoPDA@MB vesicles retained their potency in spheroids from two of the cell lines used in this study (the HCT116 and MCF7 cell lines). These results are particularly interesting, considering that 3D spheroids represent, compared to 2D cell lines, a more realistic model for preclinical drug testing and the development of classic antineoplastic drugs and PS for PDT, resembling the conditions of the cells in their in vivo environment [60,61].

The types of cell death induced by PDT have been categorized mainly into apoptosis (type I), autophagy (type II), and necrosis (type III), although the cytoprotective or cytotoxic role of autophagy is still debated; nevertheless, other cell death mechanisms have been discovered over the years, including necroptosis, ferroptosis, and mitotic catastrophe [8,9]. Previous works have shown that apoptosis could not be the predominant process that mediates cell death induced by PDT, but only a by-product of other activated mechanisms [9]. Furthermore, besides evidence indicating that MB induces both apoptosis and necrosis [47], other authors observed that the predominant type of cell death following PDT with MB depends on the protocol adopted. Moreover, there may be variations from apoptosis to necrosis depending, for example, on the energy dose used [47,62]. In the conditions employed in this work, when MB was used as a free PS and PDT was performed, necrosis was largely induced, while lipoPDA@MB tended to mainly induce apoptosis. Interestingly, the same behavior was observed in both monolayer and 3D-cultured cell lines.

It is generally acknowledged that both type I (ROS production) and type II (singlet oxygen production) photodynamic reactions are implicated in the tumor cell response to PDT and that the production of these bioactive compounds correlates with successful PDT [9]. Our results indicate a higher involvement of type I photodynamic reactions compared to type II ones, despite others who have previously shown that both ROS and ^1^O_2_ are generated by MB [41,63]. However, these results could be explained, considering that several factors may influence the ratio between type I and type II reactions, such as the type of sensitizer, the concentrations of substrate and oxygen, as well as the binding affinity of the sensitizer for the substrate [3]. Interestingly, lipoPDA@MB vesicles were significantly more proficient in inducing ROS production in all the cell lines, thus adding a further explanation for their better photodynamic performances.

Notably interesting are the results obtained on the MDA-MB231 cells. This cell line is often used as a model of triple-negative breast cancer (TNBC), which represents a particularly incurable and deadly class of tumors. Thus, finding effective new drugs or therapeutic modalities is fundamental because TNBC tumors are the greatest challenge in breast cancer treatment nowadays [64,65]. We have shown that lipoPDA@MB vesicles are even more effective on MDA-MB231 cells than on the ER-positive MCF7 cells, as evidenced by the significantly lower IC_50_ value obtained. This higher sensitivity could result from the higher apoptotic cell death induction, probably due to the higher uptake and ROS level production observed following PDT with lipoPDA@MB. Furthermore, it has been reported that TNBC cells present low intracellular GSH levels compared to ER-positive and normal cells [62,66]. This can lead to a higher sensitivity of the former to PDT-induced oxidative stress. These results indicate lipoPDA@MB-PDT as a potential treatment for cells that lack specific therapeutic targets by impacting metabolic properties that differ from those found in normal tissues.

## 4. Materials and Methods

### 4.1. Reagents and Chemicals

Water, cholesterol (chol), the grade salts for phosphate-buffered saline solutions (PBS), 2-(3,4 dihydroxyphenyl)ethylamine hydrochloride (dopamine), potassium chloride, glucose, sucrose, Sephadex G50 medium, methylene blue (MB), and ethanol were from Merck Italy (Merck Life Science s.r.l., Milan, Italy). Lipoid E80 (LE80, egg yolk phosphatidylcholine ≥ 80%) was from Lipoid (Lipoid, Ludwigshafen, Germany). 1,2-dioleoyl-sn-glycero-3-phosphoethanolamine-N-(lissamine rhodamine B sulfonyl) (ammonium salt, 18:1 Liss Rhod-PE) was from Avanti Polar Lipids (Avanti Polar Lipids, Inc., Birmingham, AL, USA).

All other reagents and chemicals, unless otherwise indicated, were purchased from Euroclone (Milan, Italy).

### 4.2. Preparation and Characterization of Liposomes

PDA-coated liposomes (lipoPDA) were prepared to employ a two-stage approach, as previously reported, with slight modifications [42,67]. Firstly, liposomes with composition LE80 (10 mg·mL^−1^) and cholesterol (10% molar ratio) were obtained through the Micelle-To-Vesicle Transition (MVT) method. For this purpose, the lipid mixture in chloroform solution was initially dried using a gentle nitrogen flux. Then, the organic solvent was completely removed under vacuum conditions. The obtained dried lipid film was hydrated with sodium cholate (4% *w*/*w*) in phosphate buffer pH 8.0 (KH_2_PO_4_ 3 mM, 46.9 K_2_HPO_4_ mM, KCl 7 mM), vortexed, and sonicated to obtain mixed micelles. Afterward, 0.5 mL of mixed micelles were loaded onto a sized exclusion chromatographic column (SEC) to induce the transition of the mixed micelles into liposomes. Resin Sephadex G50 medium was used as a stationary phase and phosphate buffer pH 8.0 as an eluent. Thus, 1 mL of liposomes were collected after 1.5 mL of death volume. The coverage of the liposomes with the PDA was carried out by incubating the liposomal suspension with the dopamine (DA) under the following conditions: liposomes 10 mg·mL^−1^, DA 1.2 mg·mL^−1^, pH 8.0, 35 °C, 20 h, under stirring [42]. The purification of the vesicles from unreacted DA was realized through dialysis (cut off 14,000 Da) against phosphate buffer pH 7.0 (KH_2_PO_4_ 17.9 mM, K_2_HPO_4_ 32 mM) for 24 h at room temperature. Fluorescent vesicles for cellular uptake experiments (Rhodamine-lipoPDA) were prepared by adding 18:1 Liss Rhod-PE (0.3% *w*/*w*) to the organic lipid blend.

MB-loaded lipoPDA vesicles (lipoPDA@MB) were obtained by adding 100 μL of MB solution (12.5 mM) in phosphate buffer pH 7.0 to 1 mL of lipoPDA vesicles and leaving them to interact for 1.5 h at 25 °C, under stirring. The purification of the liposomes from non-adsorbed MB was carried out by size-exclusion chromatography (SEC, using resin Sephadex G50 medium, phosphate buffer pH 7.0). The amount of the MB adsorbed to the liposomes was quantified through UV-Vis spectroscopy, upon extraction of MB from the lipoPDA@MB with ethanol. To this end, 100 μL of lipoPDA@MB suspension was treated with 1 mL of EtOH and vigorously vortexed. In these conditions, the MB desorbed from the PDA. The mixture was centrifuged (10 min, 10,000 rpm) to remove aggregated lipoPDA, and the supernatant was recovered. The procedure was carried out twice and the supernatants were mixed and dried under vacuum conditions. The extract was redispersed in 1 mL of EtOH and analyzed at 665 nm with a spectrophotometer (Cary 5000 UV-Vis-NIR, Agilent, Santa Clara, CA, USA) for MB quantification. The Encapsulation Efficiency (*EE*%) and Loading Capacity (*LC*%) values were evaluated as in the following equations:

$$EE\%=\frac{{mg}_{adsorbed\;MB}}{{mg}_{total\;MB\;added}}\times 100$$

$$LC\%=\frac{{mg}_{adsorbed\;MB}}{{mg}_{Lipo@PDA}}\times 100$$

The hydrodynamic diameter of the vesicles in phosphate buffer (pH = 7.0) was evaluated by Dynamic Light Scattering (DLS) analysis (Nanosizer ZS, Malvern Instruments, Malvern, UK). ζ-potential measurements were performed with the same instrument on vesicles diluted in distilled water (1:20, pH 7.0) by means of the laser doppler electrophoresis technique. Morphology of liposomes was assessed by transmission electron microscopy (TEM). Micrographs were acquired after negative staining of vesicles with uranyl acetate 1% using a JEM 1400 microscope (JEOL, Tokyo, Japan).

### 4.3. In Vitro Release

The in vitro release of MB from lipoPDA@MB vesicles was assessed through a dialysis-based method. In detail, 1 mL of the sample was placed into a dialysis tube (12,400 Da MWCO) and the tube was immersed into 14 mL of PBS 1X (NaCl 137 mM, KCl 2.7 mM, Na_2_HPO_4_ 10 mM, KH_2_PO_4_ 1.8 mM, pH = 7.4). The released MB was monitored throughout the assay by collecting 400 μL of release medium at predetermined time points and replacing the withdrawn volume with fresh buffer. The quantification of the released MB was performed through fluorescence spectroscopy (λ_ex_ 665 nm, λ_em_ 685 nm, Cary Eclipse fluorescence spectrometer, Agilent, USA), and the cumulative release (*Q*%) was evaluated as follows:

$$Q\left(\%\right)=\frac{C_{n}\times V_{t}+\sum_{i=1}^{n}C_{n_{i}-1}\times V_{a}}{Q_{t}}$$

where *Q* is the amount of MB released, *C_n_* is the concentration at the selected time point, *V_t_* is the total volume of the medium, *V_a_* is the volume of the collected sample at each pre-determined time point, and *Q_t_* is the initial amount of MB adsorbed onto the liposomes.

### 4.4. Photobleaching Assay

The rate of absorbance decay for MB and lipoPDA@MB, due to white light exposure, was determined through the photobleaching assay. Compounds were diluted in PBS, to obtain 10 μM final concentration solutions, and irradiated using a tungsten halogen light (500 W, irradiance 22 mW/cm^2^) up to 90 min; every 15 min, an aliquot was collected from each sample and analyzed spectrophotometrically at the lambda max (660 nm). The photodegradation percentage was calculated at each time point as the ratio of absorption intensity to absorption measured at the t0.

### 4.5. Biological Studies

#### 4.5.1. Cancer Cell Lines and In Vitro Culture Conditions

All the cell lines were originally obtained from ATCC (American Type Culture Collection, Manassas, VA, USA) and maintained under standard culture conditions (37 °C; 5% CO_2_). HCT116 and HT29 cells (colon cancer cell lines) were maintained in DMEM medium, while MCF7 and MDA-MB231 (breast cancer cell lines) cells were cultured in RPMI1640 medium. The WH1 human fibroblast cell line was maintained in ISCOVE medium. All mediums were supplemented with 10% fetal calf serum, 1% glutamine, and 1% antibiotic mixture; an extra 1% sodium pyruvate and 1% non-essential amino acids were added in DMEM.

To produce the corresponding spheroids, HCT116 and MCF7 cells were detached, and 2.5 × 10^3^ cells/well were then seeded onto 96U plates Nunclon Sphera (Thermo, Milan, Italy) and incubated at 37 °C in a 5% CO_2_ atmosphere. Spheroids were used on day 7 after seeding.

#### 4.5.2. Effects on Cell Viability and Cell Growth

The phototoxic effect of MB and lipoPDA@MB was evaluated by the MTT ([3-(4,5-dimethylthiazol-2-yl)-2,5-diphenyltetrazolium bromide]) assay, as previously reported [68,69]. Specifically, cells were seeded into 96-well plates (3 · 10^4^/^mL^) and allowed to grow for 48 h before PS treatment (0.05 to 5 µM). After 24 h, fresh PBS replaced the drug-containing medium, and cells were irradiated under visible light for 1 h using a 500 W tungsten halogen lamp with a light irradiance of 22 mW/cm^2^ and a fluence of 100 J/cm^2^. To maintain a temperature of approximately 37 °C, a cooling apparatus, consisting of a flowing water filter, was placed between the light source and the plate containing the cells. At the end of the irradiation period, the cells were incubated in the dark at 37 °C in a drug-free medium. Then, 24 h later, the MTT assay was performed and optical densities were measured at 570 nm using an iMark Reader (BIORAD Instruments, Hercules, CA, USA). In control samples, PS treatment was omitted. The cytotoxic effects of the compounds were quantified by calculating IC_50_ values, based on non-linear regression analysis of dose-response data, performed using the Calcusyn 2.0 software (Biosoft, Cambridge, UK).

Possible not-photo-induced effects (i.e., intrinsic cytotoxic effects) of the formulations were assessed on control cultures kept in the dark and treated as described above.

The phototoxic effects of MB and lipoPDA@MB on HCT116 and MCF7 spheroids were assessed based on the evaluation of the spheroids’ growth through a dye exclusion assay. Briefly, spheroids were treated with concentrations of the two formulations corresponding to the IC_50_ values obtained by the MTT assay on 2D-cultured HCT116 and MCF7 cells. Then, 24 h later, the drug-containing medium was discarded and the spheroids were irradiated for 1 h in PBS, as described above, and incubated in drug-free medium for 72 h. At the end of this period, 3/5 spheroids for each treatment were collected independently, disaggregated using trypsin-EDTA solution, and any live cells were counted using a Burker hemocytometer, following Trypan Blue staining. Control spheroids were treated only with culture medium and incubated/irradiated as the PS-treated ones. Pictures of the same spheroids were taken through a camera connected to an Olympus IX8I microscope immediately before (t0) and at the end of treatment (t72).

#### 4.5.3. Intracellular Accumulation of LipoPDA Vesicles

Intracellular accumulation of the lipoPDA vesicles was evaluated in all cell lines following 24 h incubation with Rhodamine-lipoPDA vesicles (30 μg/mL), MB, and lipoPDA@MB (100 nM) through cytofluorimetric analysis, exploiting the rhodamine or MB fluorescence. As a negative control, cells were also incubated with lipoPDA. At the end of the exposure time, treated cells were detached using a trypsin-EDTA solution, washed in ice-cold PBS, resuspended in PBS, and analyzed with a FACSCalibur flow cytometer (Becton Dickinson, Mountain View, CA, USA). Data were processed using CellQuestPRO 5.1 software (Becton Dickinson).

#### 4.5.4. Diffusion of lipoPDA Vesicles inside Spheroids

Spheroids were obtained as reported and incubated with Rhodamine-lipoPDA. After 24 h, fluorescence distribution into spheroids was evaluated by confocal microscopy. Spheroids were transferred from 96-well plates to a microscope slide, washed with PBS, and directly observed under a Leica SP5 Confocal Microscope. The images of the equatorial plane of the spheroids were acquired and analyzed by randomly drawing 15 radial lines on the image of the equatorial plane and recording the fluorescence at each pixel.

#### 4.5.5. Evaluation of Apoptotic and Necrotic Cell Death

The percentages of apoptotic and necrotic cells were evaluated by flow cytometric analysis following staining with propidium iodide. Cells were seeded in 6-well plates (HCT116: 2 · 10^5^/well; HT29, MCF7, and MDA-MB231: 3 · 10^5^/well) and allowed to attach and grow for 48 h in a CO_2_ incubator at 37 °C before treatment with MB and lipoPDA@MB vesicles at their respective IC_50_ values. After 24 h, cells were irradiated (500 W; fluence of 100 J/cm^2^) and incubated for 24 h in the dark at 37 °C in drug-free medium. To assess the percentage of apoptotic cell death, at the end of treatment, cells were harvested, washed in PBS, and fixed in 70% ethanol at −20 °C for at least 45 min. After a further wash in PBS, DNA was stained with a solution of PI/RNAse (50 µg/mL/30 U/mL) in PBS at room temperature for 15 min. Samples were then analyzed through a FACScalibur Becton Dickinson flow cytometer equipped with an air-cooled argon ion laser (15 mW, 488 nm), using CellQuestPRO 5.1 software. The fluorescent emission of PI was collected through a 575 nm band-pass filter and the percentage of apoptotic cells in each sample was determined based on the sub-G1 peaks detected in monoparametric histograms acquired in log mode. Evaluation of apoptotic cells from spheroids was performed following the same protocol after spheroid disaggregation.

Necrotic cells were detected by omitting the fixation step in the previously described procedure. In this way, PI will enter only membrane-damaged (i.e., necrotic) cells.

#### 4.5.6. Evaluation of Intracellular Levels of ROS and Singlet Oxygen

The intracellular generation of ROS and singlet oxygen following treatment with the photosensitizer were evaluated by exploiting the fluorescence derived from the reaction between 2,7-dichlorodihydrofluoresceindiacetate (H2DCF-DA) and ROS and that of SiDMA and ^1^O_2_, respectively. H2DCF-DA easily diffuses into cells, where it is hydrolyzed by intracellular esterases and oxidized by ROS to 2′,7′-dichlorofluorescein (DCF), an impermeable highly fluorescent compound. Thus, the fluorescence generated is directly proportional to ROS levels. Si-DMA is a far-red fluorescence probe [70], composed of silicon-containing rhodamine (chromophore) and anthracene moieties (^1^O_2_ reactive site), which selectively detect ^1^O_2_. As a matter of fact, in the presence of ^1^O_2_, the fluorescence of Si-DMA increases due to endoperoxide formation at the anthracene moiety.

For both analyses, cells were seeded in 12 well plates (6 · 10^4^ cells/well) and treated 48 h later with MB and lipoPDA@MB at equitoxic concentrations corresponding to their respective IC_50_ values. Following 24 h incubation, cells were detached, washed in PBS, resuspended in an H2DCF-DA (10 µM) in PBS (for ROS evaluation) or Si-DMA (40 nM) in HBSS (for singlet oxygen evaluation) solution, and incubated at 37 °C in the dark for 45 min. After this period, samples were irradiated for 2 min, and fluorescein or rhodamine fluorescence was measured by a FACSCalibur flow cytometer through a 530 nm or 575 nm band-pass filter and intracellular ROS or ^1^O_2_ generation were quantitated in arbitrary units based on the median fluorescence intensity (MFI) using CellQuestPRO 5.1 software. Positive controls were also included, in which 3 µL of 30% hydrogen peroxide were added to a control sample (ROS positive control) or cells were incubated with 5-aminolevulinic acid for 4 h prior to incubation with siDMA (^1^O_2_ positive control).

## 5. Conclusions

Several challenges need to be addressed to improve the efficacy, safety, and selectivity of PSs to increase the clinical use of anticancer PDT. Concerning the first topic, fluorescent dyes such as MB have been repositioned as PSs; however, the hydrophilic nature of some of them, including MB, might pose a limit to their clinical efficacy in PDT. Recently, combining PDT with nanomedicine has led to promising improvements for targeted drug delivery, increasing the chance of using MB as a PS. In particular, liposomes have shown great results in drug delivery, with several approved liposomal drugs on the market in different fields, including PDT. Nevertheless, plain liposomes still have limitations that hamper their clinical use and that need to be addressed, such as encapsulation efficiency, stability in biological fluids, recognition by the immune system, and targeted delivery issues. Furthermore, research on tissue penetration and the efficacy of liposomal drugs is needed.

The liposome/PDA-based system we have realized, besides increasing the potency of MB in PDT, offers the further advantage of being able to incorporate other bioactive compounds into the lipid bilayer and/or aqueous core, thus representing a potential poly-drug delivery system, leading to enhanced cellular uptake and efficacy. Furthermore, the PDA coating could provide additional intriguing properties to the proposed carriers, such as stability in biological fluids, controlled drug release capacity, and also active targeting potential, thanks to the moieties that the polymer exposes on their surface. This makes it simple to functionalize with specific ligands for the desired cellular targets, thus enhancing its potential use [67].

## Figures and Tables

**Table 1:** Colloidal characterization of the liposomes. The errors represent the experiment’s standard deviation (S.D.) (n = 3).

Sample	Mean Diameter * (nm)	PDI	ζ-Potential (mV)
Uncoated liposomes	25 ± 3	0.18 ± 0.07	−26 ± 3
lipoPDA vesicles	53 ± 1	0.223 ± 0.006	−17 ± 2
lipoPDA@MB vesicles	63 ± 1	0.198 ± 0.007	−11 ± 1

* Based on DLS measurements.

## Data Availability

All data are available under request.

## Supplementary Materials

The following supporting information can be downloaded at: https://www.mdpi.com/article/10.3390/ijms25063392/s1.
