# Supplementary material for: Polydopamine-Coated Liposomes for Methylene Blue Delivery in Anticancer Photodynamic Therapy: Effects in 2D and 3D Cellular Models
Source: Int J Mol Sci. 2024 Mar 16;25(6):3392. doi: 10.3390/ijms25063392 (PMC10970349; doi:10.3390/ijms25063392)
Supplement: Supplementary file 1 [file ijms-25-03392-s001.zip › ijms-2887580-supplementary.pdf]

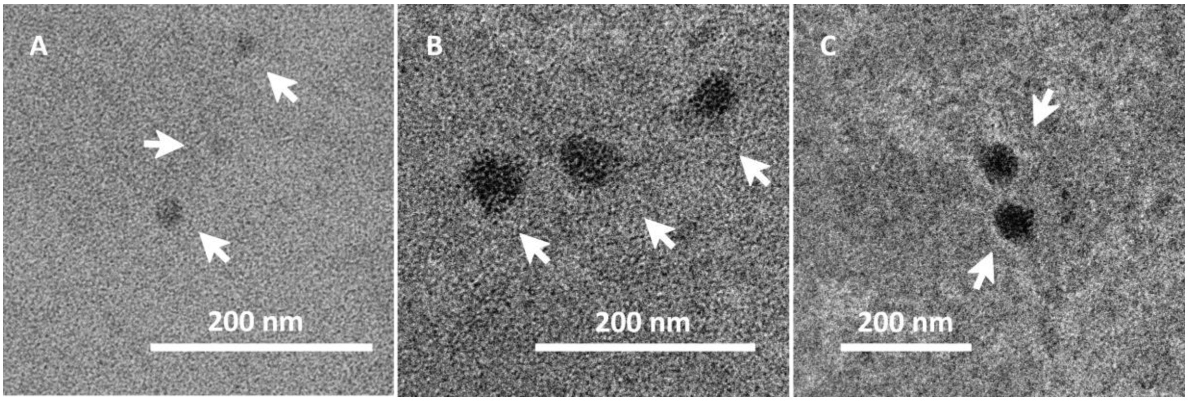

S1. TEM micrographs of (A) naked liposomes, (B) **lipoPDA** and (C) **lipoPDA@MB** vesicles

dark

IC_{50} (µM)	HCT116	HT29	MCF7	MDA-MB231
**lipoPDA**	> 5	> 5	> 5	> 5
**MB**	2,63 ± 0,37	3,6 ± 0,30	2,57 ± 0,27	3,88 ± 0,53
**lipoPDA@MB**	2,01 ± 0,19°	1,04 ± 0,21°°°	1,56 ± 0,02°	2,60 ± 0,08°

light

IC_{50} (µM)	HCT116	HT29	MCF7	MDA-MB231
**lipoPDA**	> 5	> 5	> 5	> 5
**MB**	0,403 ± 0,04***	3,4 ± 0,27	1,07 ± 1,65**	0,69 ± 0,06***
**lipoPDA@MB**	0,19 ± 0,01***/°	0,43 ± 0,09**/°°°	0,43 ± 0,07***/°°	0,33 ± 0,03***/°°°

Table S1 and S2. IC_{50} values obtained in HCT116, HT29, MCF7, and MDA-MB231 cell lines following 24h treatment with **MB**, **lipoPDA** and **lipoPDA@MB**, 1h irradiation, 24h incubation in drug-free medium and MTT assay (mean ± S.D of 3-4 independent experiments; °p<0.05, °°p<0.01, °°°p<0.001 vs **MB** same condition; **p<0.01, ***p<0.001 vs MB same condition and **MB** or **lipoPDA@MB** dark)

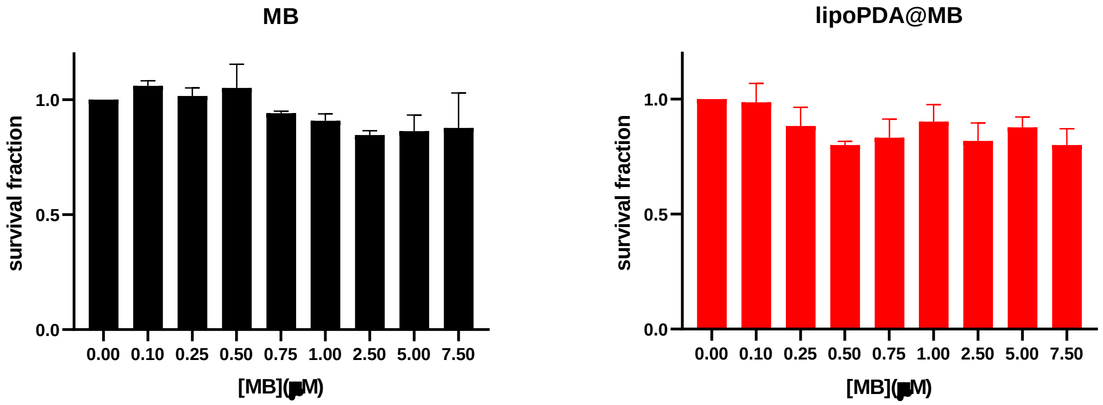

S2 – Survival fractions obtained in WH1 fibroblast cell line following 24h treatment with **MB** and **lipoPDA@MB**, 1 h irradiation, and 24h incubation in drug-free medium in the dark. (mean ± S.D. of 3 independent experiments).

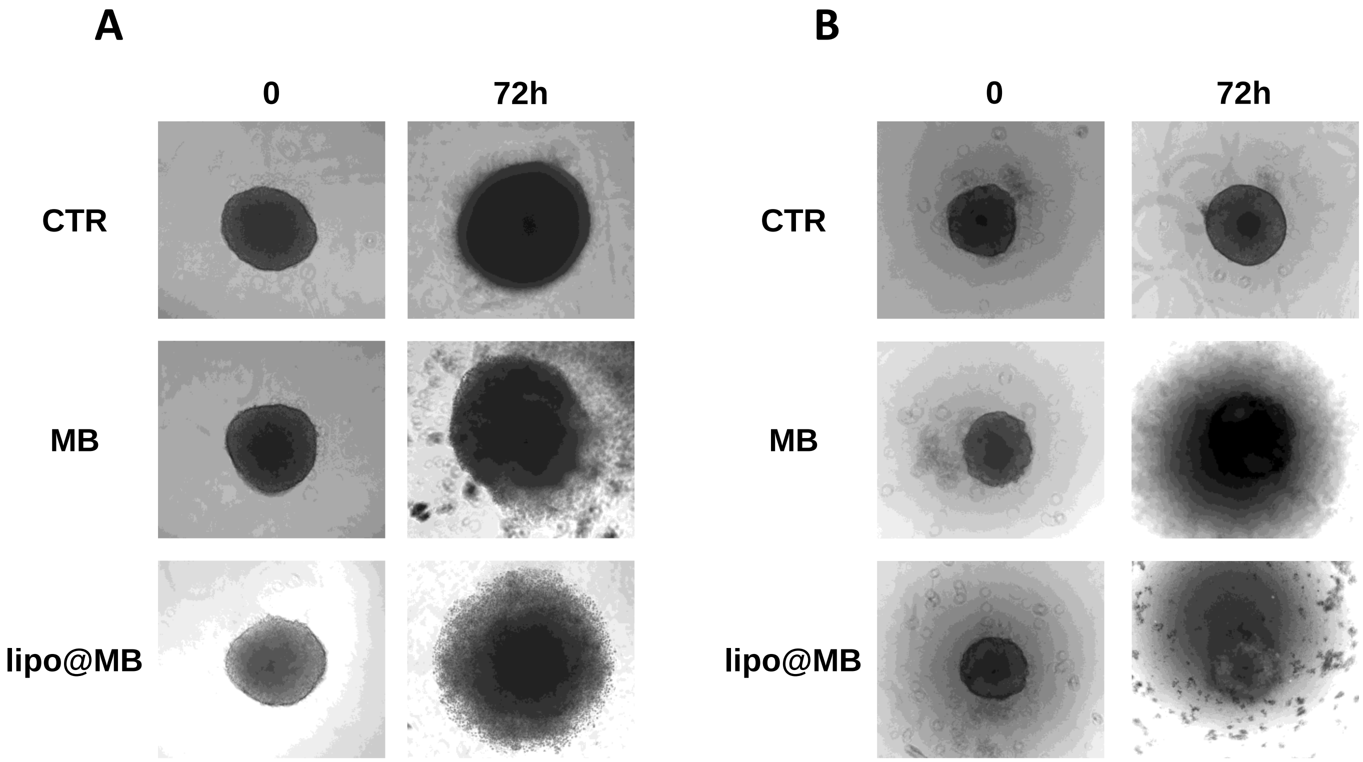

S3 – HCT116 (A) and MCF7 (B) spheroids following 24h treatment with **MB** and **lipoPDA@MB** at concentrations corresponding to the IC_{50} values obtained in monolayer-cultured cells, 1 h irradiation, and incubation in drug-free medium in the dark. Pictures of spheroids were performed immediately after irradiation (time 0) and 72 h later.

Sample	Mean diameter (nm)	PDI	ζ-potential (mV)
**Rhodamine-lipoPDA**	52.3 ± 0.6	0.211 ± 0.010	- 13.8 ± 1.7

Table S3. Colloidal characterization of the **Rhodamine-lipoPDA** vesicles

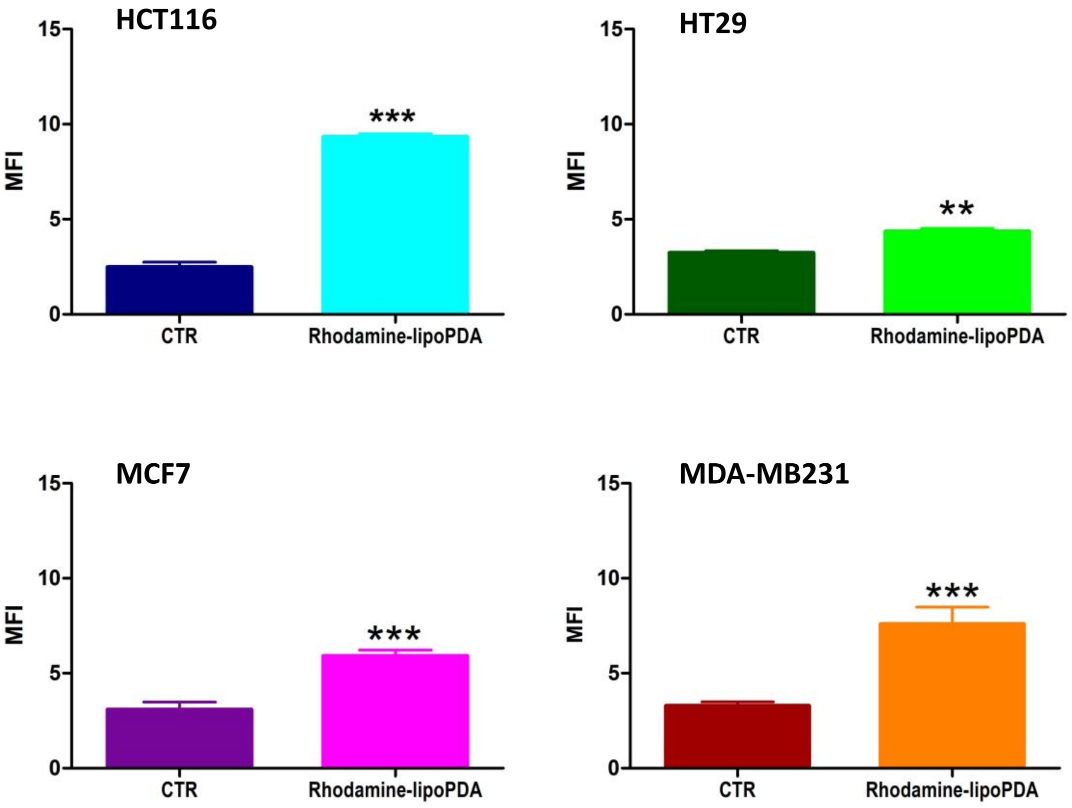

S4. Intracellular accumulation of **Rhodamine-lipoPDA**. (mean ± S.D. of 3 independent experiments; **p<0.01 and ***p<0.001 vs controls).

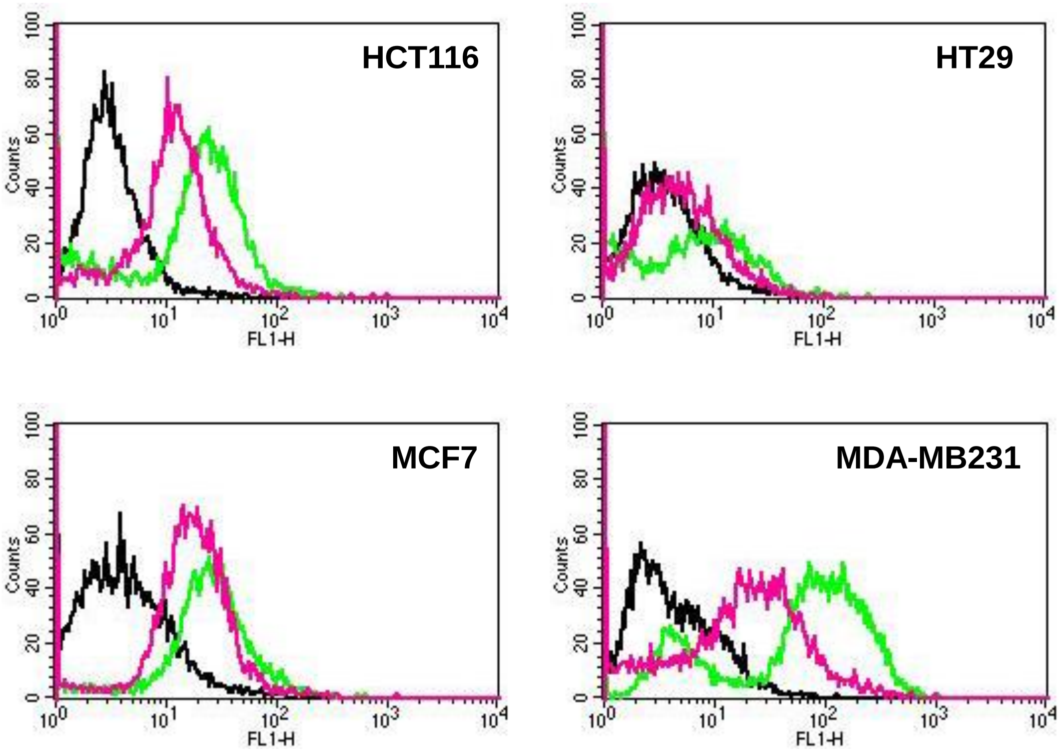

S5 – Representative images of ROS levels in HCT116, HT29, MCF7, and MDA-MB231 cells following 24h treatment with **MB** and **lipoPDA@MB** at concentrations corresponding to the respective IC_{50} values, 45′ incubation with H2DCF-DA (10 μM), 2 min irradiation, and flow cytometry analysis (black line: CTR; pink line: **MB**; green light: **lipoPDA@MB**).
